# Development and Validation of Dummies and Human Models Used in Crash Test

**DOI:** 10.1155/2018/3832850

**Published:** 2018-11-13

**Authors:** Tao Xu, Xiaoming Sheng, Tianyi Zhang, Huan Liu, Xiao Liang, Ao Ding

**Affiliations:** ^1^School of Mechanical Science and Engineering, Jilin University, Changchun, China; ^2^Tianjin Aerisafety Science and Technology Co. Ltd, Tianjin, China

## Abstract

The crash test dummy, an important tool for car crash safety tests, is of great significance to explore the injury biomechanics of the occupants and improve the safety performance of the vehicle. The article mainly consists of four parts: brief introduction of injury mechanism, early experiments for obtaining biomechanical response (animal tests, cadaver tests, and volunteer tests), and development and validation of mechanical dummies and computational models. This study finds that the current crash test dummies are generally designed based on European and American, so they have limitations on the damage prediction of other regions. Further research in the crash test dummy needs the participation of various countries in order to develop a crash test dummy that meets the national conditions of each country. Simultaneously, it is necessary to develop dummies of vulnerable groups, such as the elderly dummy and obese people dummy.

## 1. Introduction

Automobiles provide great convenience and quickness for people's life and make a great contribution to the economy and social development. However, with the rapid development of the automotive industry, road accidents have also suddenly increased, resulting in a large number of casualties and economic losses. According to the World Road Safety Global Status Report on Road Safety 2015 issued by the World Health Organization, it can be seen that the number of people who died from traffic accidents is roughly around 1.25 million every year, which means that one person is killed in traffic accidents every 25 seconds on a global scale. Traffic accidents are the main cause of death in the 15- to 29-year-old population [1]. China, the most populous country in the world, had a total of 187,781 road traffic accidents in 2015 [2]. The total direct property losses caused by the accident were 103,692 million yuan, of which the total number of injured people w s 199,880, and the death toll was 58,022. These shocking figures all indicate that improving the safety protection for occupants and reducing the casualties caused by traffic accidents have become an important issue to be solved urgently.

In fact, as early as the 1950s, in order to investigate the human injuries caused by collisions and correctly assess the actual injuries suffered by occupants in car collisions, researchers began to study the injury biomechanics of occupants in car collisions. Researchers used human corpses as surrogates to collect data on human injuries caused by accidents in crash experiments and subsequently adopted animals and volunteers as crash surrogates. Although these experiments provided valuable data for collision safety, they were gradually abandoned due to restrictions such as ethical and moral constraints, physiological function differences, experimental risks, and experimental irreproducibility. With the development of science and technology, crash detection device—mechanical crash test dummy, known as anthropometric test device (ATD), came into being. When dummy is subjected to physical quantities such as force, acceleration, and speed during a car crash, the mechanical response curve should be highly fitted to the data obtained from human cadaver experiments. Using the crash test dummy to perform repeated crash tests, the injury location can be effectively predicted, and the injury indicators of the occupants can be estimated.

This paper reviews the development of dummies and is introduced from four aspects. The first is theoretical foundation of crash test dummy, named biomechanical injury mechanism. In order to study the injury mechanism, researchers conducted cadaver experiment, animal experiment, and volunteer test in early time, so the second aspect is about biomechanical test. The third and fourth aspects introduce the development and verification process of the mechanical dummies and computational models used in crash test.

## 2. Injury Criteria

The main purpose of crash test dummy used as a substitute for human in car collisions is to determine the injury severity to human body caused by the accident. Thus, understanding how the mechanical properties of dummy meet injury mechanism of human and correspond to the harm standard is absolutely essential. The current study believes that the blunt impact injury mechanism is the degree of deformation or strain of the tissue exceeding its recoverable limit [3]. In the car crash, the main load type of human exposure to injury is blunt impact. The main sites of injury are the head, neck, chest, abdomen, pelvis, and other parts of the extremities. In order to describe the human injury condition intuitively, according to the type of injury of the human body when it is damaged by impact, the corresponding injury index is formulated.

The Abbreviated Injury Scale (AIS) (shown in Table 1), proposed by the Association for the Advancement of Automotive Medicine (AAAM), standardized the injury types and ranked injury levels by severity. It is the most widely used measurement for crash injury currently. However, the dummy can only output parametric impact result rather than the visualized injury characterization. Therefore, it is important to seek the relationship between assessment of human injury by severity and loads on the dummy. Researchers fitted the risk assessment equation of the corresponding injury site through a large number of accident statistics and converted the experimental data into the corresponding injury types and severity in reality (shown in Table 2).

In car collisions, most of the deadly head injuries come from the impact fracture of the skull and the brain tissue injury. National Highway Traffic Safety Administration (NHTSA) raises the HIC value based on the acceleration to measure the max limit of injury to the human head in the collision of the car. The widely used HIC value is calculated by (1). The formula is as follows:

(1) $$\begin{array}{c} HIC=\left(t_{2}-t_{1}\right){\left[\frac{1}{t_{2}-t_{1}}\int_{t_{1}}^{t_{2}}adt\right]}^{2.5}, \end{array}$$

where *t*_1_ and *t*_2_ (s) are two time points in the crash acceleration curve. *a* is measured as a multiple of the gravitational acceleration (*g*), and the equation uses a three-way synthetic acceleration. It also stipulates that the time difference between *t*_1_ and *t*_2_ cannot exceed 36 ms; HIC value cannot exceed 1000 (tolerance limit). Hertz [4] fitted the relationship between HIC and the probability of skull fracture (AIS ≥ 2) by experimental data and found that for 50th male, the probability of skull fracture was about 48% when HIC is 1000.

Neck injury has become the most frequent injury in car crash accident and also one of the most important causes of occupant's disability. NHTSA [5] proposes the guideline *N*_*ij*_ to evaluate the neck injury in frontal impact car crash. *N*_*ij*_ was defined by neck axial force *F*_*z*_ and force moment *M*_*y*_. The formula is shown as follows:

(2) $$\begin{array}{c} N_{ij}=\frac{F_{z}}{F_{\mathrm{int}}}+\frac{M_{y}}{M_{\mathrm{int}}}. \end{array}$$

The *N*_*ij*_ value can be used to estimate the neck injury on AIS1 level. Bohmann et al. [6] studied the neck injury on AIS1 and claimed that the tolerance limit should decrease to 0.2 and 0.16 for long term and short term damage, respectively.

When the chest suddenly is decelerated due to blunt instrument impact, the injury mechanisms include three main types: compression, viscous loads, and inertial loads of internal organs. Injury results can be categorized as skeletal injury and soft tissue injury. In general, the main forms of injury are rib fractures and lung injuries, as well as a smaller chance of heart bruises and ruptures and rupture and breakage of the aorta. The chest composite index represents a chest injury criterion in frontal impact. The response under compression coupled with acceleration is considered. At the same time, the load of the airbag to the occupant and the restraint effect of the seatbelt to the occupant are described. The definition of CTI is evaluated by a combination of the 3 ms resultant acceleration of the spine and the amount of deformation of the chest. The CTI value is calculated as follows:

(3) $$\begin{array}{c} CTI=\frac{A_{\max}}{A_{\mathrm{int}}}+\frac{D_{\max}}{D_{\mathrm{int}}}, \end{array}$$

where *A*_max_ is the single peak value (*g*) of 3 ms for the resultant acceleration of the spine; *A*_int_ is the 3 ms intercept reference (*g*); *D*_max_ is the maximal chest deformation (mm); and *D*_int_ is the intercept reference value (mm) of the deformation.

The abdomen peak force (APF) was elaborated by European ECE R95 guideline and rules that the external force of abdomen should not exceed 4.5 kN.

The injury mechanism of femoral fractures caused by collisions with dashboards, which often occurs in frontal crashes in cars, is mostly caused by axial compression (62%), followed by bending (24%), twisting (5%), and shear (5%). Because the femur is not completely straight, the shape of the femur will affect the fracture in the case of indirect loading. Similar to fractures of the femur, tibial fractures can also be caused by retrograde direct or indirect loads. Pubic symphysis peak force (PSPF) in ECE R95 rules that the collision force at the pubic symphysis should be less than 6 kN. The criteria for tibial fractures, also known as the tibial index, are used to evaluate the tibia injuries. It is calculated by the hinge restraint of the fixed hinge on the load sensor at the upper and lower positions of the sacrum, as defined by each force and moment value.

(4) $$\begin{array}{c} TI=\frac{M_{R}}{M_{R\text{MAX}}}+\frac{F_{Z}}{F_{Z\text{MAX}}}, \end{array}$$

where *F*_*Z*_ refers to the axial pressure of the lower leg (kN); *F*_*Z*MAX_ refers to axial pressure threshold; $M_{R}=\sqrt{M_{X}^{2}+M_{Y}^{2}}$; *M*_*X*_ and *M*_*Y*_ refer to bending moment of *X* and *Y*; and *M*_*R*MAX_ represents the synthetic bending moment threshold.

## 3. Biomechanical Tests in Early Time

To improve the car's ability to protect the occupant and reduce human injury during car collision, it is necessary to have a preliminary understanding of the occupant's biomechanical response during the collision. In the early stages, there are three kinds of biomechanical tests to explore biomechanical responses: volunteer tests, animal tests, and human cadaver tests.

In the field of volunteer test, US Air Force Colonel John P. Stapp is a well-known pioneer. He personally went through a series of tests and even sat on a rocket skateboard with a speed of up to 1000 km/h. His volunteer tests obtained valuable data which were later widely used in the injury biomechanics, such as human body acceleration tolerance data [7]. However, crash tests have certain risks, and volunteer tests are inevitably performed at low-speed and light-load conditions, such as head injury study at low rotational speeds [8] and spine deformation study at low-speed rear impacts [9]. For biomechanical studies under high-speed and heavy-load conditions, volunteer tests are obviously not suitable.

In order to study the physiological responses under heavy load conditions, some scholars conducted experiments on living animals. In 1980, Ono et al. [10] conducted a head impact experiment on live monkeys and found that impact acceleration, impact contact area, and other factors will affect the head injury. When the brain of a monkey suffered a fracture, the tolerance value was at a dangerous threshold. Combining the obtained data with the results of the human cadaver skull impact test, a human head impact tolerance threshold can be deduced. In 1981, twelve anesthetized male pigs were used by Kroell et al. [11] to study the chest injury mechanism, injuries such as cardiovascular ruptures, pulmonary contusions, and skeletal fractures. The results emphasized the importance of loading speed for determining the overall severity of chest blunt impact. Although animal tests can provide a biological reflection basis, the animal body mass distribution and morphological characteristics are different from the human body. Therefore, the results of animal experiments have limited promotional value.

In general, fresh human cadaver is a better substitute for biomechanical studies of impact injury, and there are corresponding cadaveric tests to investigate the response of parts of the body (head, chest, etc.). Hodgson and Patrick [12] found that when the head of a cadaver received a sinusoidal vibration input, the mode frequency of the skull corresponds to spring-mass system. In response to this discovery, they proposed a method to compare the cadaver head response to spring-mass system. Kroell et al. [13, 14] conducted a series of tests to study the responses of cadaver's chest. 23 cadaver samples of different ages, heights, and weights were chosen to be used in tests. Impactor mass and velocity were in various combinations to apply to tests. These tests obtained valuable chest response data.

In the abovementioned volunteer tests, animal tests, and cadaver tests, there are significant drawbacks such as experimental risks, physical differences, and violating ethics. Therefore, developing a new human substitute to apply to the research on vehicle impact injury biomechanics is important. The substitute model is supposed to have the same structure, size, mass distribution, and impact motion characteristics compared to human body. The crash test dummy is such a substitute for human body in crash tests. It is made of various materials such as steel, aluminum, rubber, and polymers and is equipped with multiple acceleration sensors, force sensors, torque sensors, and displacement sensors to record responses.

## 4. Mechanical Dummies

### 4.1. Development

In 1949, the first dummy was used in the air force; after years of development, the dummies have been widely used as substitutes for human body in car crash tests. According to the type used for collision, the dummies can be categorized as frontal impact dummy, side impact dummy, and rear impact dummy.  Table 3 lists crash test dummy types and their application conditions. In order to better understand the development of mechanical dummies, the following describes in detail the development process of each series of dummy.

#### 4.1.1. Frontal Impact Dummy

In 1971, ARL and Sierra collaborated to develop the Hybrid I dummy. This dummy can be used to measure head and chest triaxial acceleration and femur load. In 1972, with the support of the U.S. automotive giants, FTSS (First Technology Safety Systems) developed the Hybrid II dummy [15]. Many parts had been redesigned to achieve better results: the head/neck interface was more anatomical, the improved neck mount model facilitated the reproducibility of head kinematics, the self-centering shoulders and improved shoulder load distribution yielded more repeatable responses, and lower torso with butyl rubber lumbar spine improved overall repeatability. In general, its major improvements over Hybrid I dummy designs were good durability and acceptable repeatability. In 1973, ATD 502 dummy was developed. By improving the material and positioning structure, this dummy achieved a more human-like seating posture and a better repeatability. Although ATD 502 dummy had made a great progress, the biomechanical responses of various parts were still lacking. In 1976, General Motors (GM) made significant improvements in the neck, chest, and knees of Hybrid II and ATD 502 to develop Hybrid III dummy, whose biofidelity and injury prediction measurement capacity had been improved. Nowadays, the Hybrid III dummy has been widely used in the field of car crash tests, including the 50th adult male dummy, the 95th adult male dummy, and the 5th adult female dummy. The Hybrid III 50th adult male dummy is currently the most widely used dummy in various countries. The Federal Motor Vehicle Safety Standard (FMVSS 208) clearly stipulates that the Hybrid III 50th dummy is designated as frontal impact dummy in car crash tests.

The THOR dummy program had been supported by National Highway Traffic Safety Administration (NHTSA) of the United States since last century. Currently, the improved THOR-M dummy has been qualified to enter the market and the Euro NCAP is considering using the THOR-M dummy for future frontal impact tests. Compared with the Hybrid III dummy, the THOR-M dummy has better damage prediction ability and has more human-like characteristics. For example, the THOR-M dummy has sensors mounted on the face to measure facial injuries in frontal crashes, while the Hybrid III dummy cannot predict such risks. Two wire spring dampers are added to the neck to simulate the head rotation lag. The flexibility of the neck is closer to human characteristics. In summary, the THOR-M dummy provides more body injury measurement data than the Hybrid III dummy and it will be widely used in the frontal impact test in the future.

#### 4.1.2. Side Impact Dummy

In the late 1970s, the University of Michigan and the NHTSA jointly developed the world's first side impact dummy SID which was developed according to 50th American male [16, 17]. Its head and neck retained the structure in Hybrid II, and foam parts are used instead of the omitted arms in the torso. The chest of the SID cannot simulate the chest response of human for its material had no elasticity in the horizontal direction.

As SID dummy developing, Europe also launched the development work of the side impact dummy. During 1978~1982, three dummies produced by APR, ONSER, and MIRA were released, respectively [18]. Although these dummies cannot obtain the desired lateral impact response, they provided prototypes for the new side impact dummy EuroSID. The EuroSID-I was developed according to the European male size in the mid-1980s.

The SID and EuroSID were evaluated by the International Standards Organization (ISO) to be found without sufficient biofidelity [19]. In response to this conclusion, a biofidelic side impact dummy named BioSID was developed by General Motors and Society of Automotive Engineers (SAE) [20]. The head, neck, shoulders, chest, abdomen, and pelvis of the BioSID have good biofidelity in side collisions. SID-IIs was developed in 1995, representing a 5th small female. In 2000, EuroSID-II (ES-2) was developed and upgraded based on EuroSID-I; a lot of changes were made in the original structure, for example, a load sensor was added to the head-neck contact surface, reducing the coefficient of friction between the clavicle and the mounting plate, added a new backplane with load cell, and so on.

In 1997, the ISO initiated the development of a more biofidelic side impact dummy: the WorldSID dummy. WorldSID dummy was based on the medium size of men worldwide. The reproducibility, the durability, and the sensitivity have been greatly improved compared to other dummies.

#### 4.1.3. Rear Impact Dummy

In the 1990s, a consortium consisted of Chalmers University, Volvo Car Corporation, and Saab Automobile AB was formed to develop the new dummy BioRID which was used in rear impact [21]. The BioRID dummy was designed to represent a 50th male in Europe, and its vertebral column curve fitted well with that of human. The vertebral column consisted of 24 separate vertebrae; the vertebral column will perform realistic movements when faced with impact load. Compared with Hybrid III dummy, BioRID dummy is more closely related to human characteristics on the neck and vertebrae [16]. Therefore, it is more realistic to simulate the human response after a rear-end collision in a rear collision accident.

It can be seen from the development of the dummies that all kinds of dummies have undergone continuous improvement, so that the response of each part of the dummy can be more and more close to the human body response. However, most of these dummies are designed based on the male size in Europe and America. But the size of the human body varies greatly from country to country. For example, the height and weight of 50th male in China were 167.8 cm and 59 kg (GB 10,000–1988), these values differ from those of Hybrid III (175.5 cm and 65.5 kg). Furthermore, the center position, moment of inertia, and radius of rotation of various parts of the human body are closely related to the height and weight of the human body. In this respect, the dummy may have limited ability to predict the injury of people who are not European and American.

### 4.2. Validation

As the key equipment for vehicle collision safety inspection, the crash test dummy must not only be similar to the human structure in terms of external dimensions and mass distribution, but at the same time, the mechanical response of the major parts of the dummy should also be highly similar to the biological response of the same part of the human body. The higher the similarity is, the easier it is to get a more accurate injury assessment. Therefore, it is very important for the artificial simulation of dummy. In different collision conditions such as frontal impact, side impact, and rear impact, the major parts of the injured parts are not exactly the same, the forms of injury are different, and the method of verifying the biofidelity of the dummy is also different. According to the type of collision, the following introduces the validation of different dummies.

#### 4.2.1. Frontal Impact Dummy

In the frontal impact, the most vulnerable parts of the body are the head, neck, chest, and knee. The Hybrid III is the most widely used frontal impact dummy around the world, and it has been done in various parts of rigorous tests to validate the biofidelity of dummy; Foster [15] detailed the validation process of the head, neck, chest, and knee. For head validation, the head was dropped from a position of 376 meters high to a flat rigid steel plate, three acceleration measurements were taken at the head center of gravity, and the acceleration directions were orthogonal to each other. The resultant of three accelerations was the final head response. For neck validation, biomechanical neck responses can be divided into response to flexion and extension tests. The whole dummy was restrained to conduct the sled tests, and the angle responses were obtained from high-speed motion pictures, while torque responses were measured by the dummy's neck load transducer. For chest validation, each dummy “sitted” on a flat surface with the upper and lower limbs and ribs parallel to the seating surface, a ballistic pendulum impactor weighing 4.3 kg struck at the center of the sternum with impact velocities 4.3 and 6.7 m/s. By multiplying the impactor mass and the deceleration, the chest impact force could be obtained. A potentiometer was used to measure the sternum relative to the thoracic spine, which was called chest deflection. For knee validation, each upper leg needed to be installed horizontally and there was an angle of 1.15 radians between the upper leg and lower leg; three pendulum impactors weighing 0.5 kg, 1.0 kg, and 1.5 kg were used to impact the knee along the axis of the femur, respectively, and the deceleration during the impact could be measured by axis accelerometer mounted on the impactor. Knee impact force was obtained from the product of pendulum mass and deceleration. The responses of the four parts of the validation were compared with the cadaver data obtained by Hubbard and Mcleod [22], Mertz et al. [23], Neathery [24], and Horsch and Patrick [25], and the responses of the Hybrid III dummy were all distributed in the range of the cadaver data.

#### 4.2.2. Side Impact Dummy

When the car is subjected to a side collision, the most vulnerable parts of the human body are the head, neck, shoulders, chest, abdomen, and pelvis, and each part needs to be validated. ISO had made a rating scale to evaluate the biofidelity of dummy as shown in Table 4. Scherer et al. [26] conducted tests according to ISO to judge the side dummies. For the head, neck, and chest validation, the test processes were similar to those of the frontal impact dummy, except that the experimental parameters were different, such as the head dropped from 200 meters instead of 376 meters, the sled used for neck validation changed to 6.9 and 5.8 m/s, and the impact direction of pendulum impactors changed. For the shoulders and pelvis, these parts are mainly affected by the blunt impact of the door; when the validation tests were conducted, rigid pendulum impactors were used to impact at certain velocities.

As can be seen from Table 5, all the side impact dummies have the acceptable biofidelity. The WorldSID performed well in many parts of biofidelity comparison, and the WorldSID is the only side impact dummy which can get “good” level from the overall performance. Most of the previous side impact tests used ES-II dummies. Now, WorldSID has become the side impact test dummy in U-NCAP, C-NCAP, and other regulations with its good biofidelity.

#### 4.2.3. Rear Impact Dummy

The validation of the BioRID was conducted by comparing the responses with the PMHS data and volunteer data. Davidsson and Linder had contributed a lot to the validation in the early time; they carried out the validation tests at different impact velocities by different impact types. For example, Linder et al. [27] conducted sled tests to evaluate the BioRID. The sled used in the tests was generated by compressed air, and the acceleration pulse of sled was controllable. When compared with PMHS data, the dummy was exposed to a change of velocity (Δ*v*) of 10 and 15 km/h, while compared with volunteer data, the dummy was subjected to a maximum acceleration of 3.5*g* at Δ*v* of 10 km/h. In horizontal accelerations and displacements of the head and the chest, the neck forces were chosen as the comparison indicators. The responses of BioRID correlated well with the volunteer and PMHS data, which indicated that the BioRID can be used as a sensitive tool for rear impact.

The neck is most vulnerable to injury when the rear-end impact occurs; some researchers focused on this part. Ono and Kaneoka [28], Davidsson et al. [29], and Geigl et al. [30] used volunteer tests to obtain data on human neck injuries in rear-end impacts. Foret-Bruno et al. [31] analyzed the injury status and the form of motion of the head and neck and the corresponding parts of the back of the Hybrid III dummy in the postimpact mode. The conclusion is that the stiffness of the neck of the Hybrid III dummy is quite different from that of the human body.

From these experimental results, the existing dummy model currently used anthropomorphic crash test dummies that can reflect the human response to a certain extent, but they are limited in their biofidelity and in their application type. Further improvement research on existing physical dummy is necessary.

## 5. Computational Models

Nowadays, commercial mechanical dummies are expensive and consume huge during crash tests. Only large corporations and research institutes have the financial resources to purchase physical dummies for research on car crash safety. With the continuous advancement of computer technology and digitization methods, visual model in computer is also widely used in automotive crash simulation. Currently, the models used for car crash studies mainly include multirigid models and finite element models. Multirigid body models are based on multibody dynamics theory. Engineers use simple planes and ellipsoids to simulate various structures of the human body and construct adult body model, using ADAMS, MADYMO, and other software to analyze. The finite element model uses the principle of finite element method to build the model. The essence of the finite element method is to discretize the entire study object. In contrast, the finite element model is more detailed so that it can investigate the local deformation and stress distribution. Therefore, the application of the finite element model is more extensive.

### 5.1. Traditional FE Models

The study of finite element dummy for car crash originated in the late 1970s. Some companies have developed recognized FE dummy models, such as ERAB, ETA, FTSS, ARUP, and FAT [32]. Based on the mechanical dummies mentioned above, the FE model of dummies can be developed by five steps [33]. Firstly, capture the geometries of mechanical dummies by 3D scan. Secondly, translate the obtained geometries to CAD data. Thirdly, represent the model with 3D elements that means generating the FEM meshes. Fourthly, develop single components. Lastly, validate the model; the validation process is consistent with that of the mechanical dummy. Recent advancements in computer hardware technology and software developments have made it possible to develop detailed finite element models, by increasing the model structural details, refining mesh density, and improving material properties to improve the calculation accuracy of FE model. Nowadays, the commercial mechanical dummies all have a finite element dummy corresponding to them; the most recognized FE models are developed by FTSS.

Many scholars also have validated the finite element dummies by comparing with physical tests or regulations. In 2002, Noureddine et al. [34] illustrated the construction and validation of the Hybrid III dummy FE model in detail. The simulation results of chest model, head model, and neck model were compared with the mechanical dummy tests according to the Code of Federal Regulations. The time histories of the chest acceleration and head acceleration showed reasonable agreement with the results of physical test. In 2007, Friedman et al. [35] performed a head drop test using a Hybrid III finite element dummy to compare the upper neck force with the test in published mechanical dummy test. The results demonstrated that FE model shows good agreement with the test response in a rollover crash environment. In 2013, Tanaka et al. [36] studied the relationship between external force to shoulder and chest injury using WorldSID FE model. According to the seating posture and impact position of the manual to perform the CAE, there was a good agreement between CAE simulation results and physical test results. In 2017, the FE model of 5th percentile THOR had been compared with biofidelity corridors from head to toe [37]. The peak thorax probe impact response can be consistent with that of biofidelity corridors.

### 5.2. Human Models

Since the 1990s, in order to study human injuries in more detail, scholars have begun to explore the biofidelic human models gradually. The human model is developed based on the human body's geometric dimensions and anthropomorphic material properties. It can predict human injuries such as skeletal fractures, internal organ injuries, stress distribution of brain tissue, and skin contusion. There are several available whole-body human models, including H-model [16], Ford human body model [38], WSU human model, HUMOS, THUMS, and GHBMC model. The latter four models are relatively widely used. The development of them is described, respectively, as follows.

**Figure 1:**
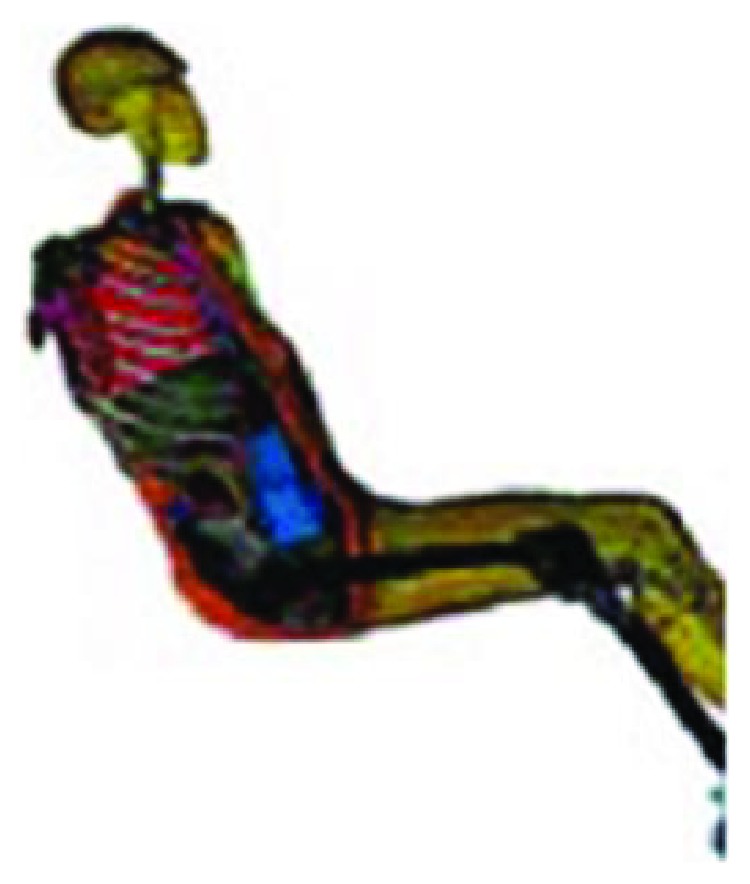
WSU model [46].

In the past 20 years, Wayne State University (WSU) Bioengineering Center has been devoted to the development of finite element models as shown in Figure 1. Since 1993, a skull-brain FE model of the human which is called the WSUBIM model was developed. The initial version of the WSUBIM model was designed to simulate the basic anatomy of the human head (including the scalp, cerebral spinal fluid, dura, parasagittal bridging, venous sinuses, three-layered skull, gray matter, white matter, cerebellum, falx, pia matter, tentorium, brain stem, and ventricles) and facilitate further study of head injury mechanisms [39]; the model was able to predict the sensitivity of the brain to the effects of impact from different directions and the location of diffuse axonal injury (DAI) in the brain. In addition, a sliding interface was added to the model to simulate the interaction between the matter and cerebral spinal fluid [40]. With the sliding interface introduced, the model was capable of predicting the relative displacement time histories of the brain. The response data could be matched with pressure and contact force data by Nahum [41]. Based on the previous work, a more detailed WSUBIM model was developed. The density of the mesh had been further improved, and the number of model elements rose from 41,354 to 314,500, when nodes increased from 32,898 to 281,800 [42]. The new detailed model has the ability to simulate at high rotational acceleration conditions up to 12,000 rad/s^2^ and has been validated against published cadaveric test data [41]. WSU also studied the other advanced models involving the human chest [43], neck [44], and abdomen [45], and their validation is confirmed by experiments conducted at the experimental center of WSU. The WSU human models have served many workers and institutions as a basis for their own development and research (Ford, General Motors, Nissan, Toyota, ESI, Mecalog, etc.).

**Figure 2:**
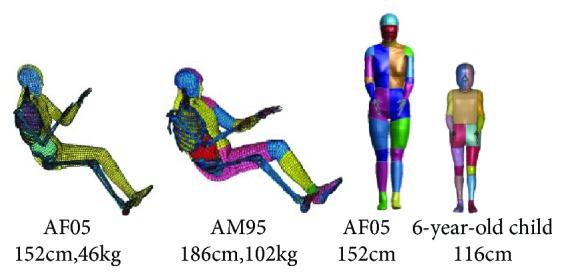
THUMS models [53].

At the beginning of the 21st century, Toyota Motor Corporation developed a new type of total body finite element dummy called THUMS [47]. According to the data obtained by Schneider et al. [48], the THUMS was first scaled to fit the 50th percentile of American male which consists of a base model and several detailed models (head/face, shoulder, and internal organs). The base model totally includes 60,000 nodes, 1000 materials, and 83,500 elements; solid elements were used to represent the spongy bone while the cortical bone was modeled using shell elements; there was a ligament connection between the bones, and sliding interfaces were defined in the contact area; the whole model had no mechanical joint [49]. Several simulations were performed to compare with the data of cadaveric test to validate impact responses of each body part [50, 51]. The model was used in injury reconstruction and successfully reproduced multiple injuries of an occupant, such as bone fractures and ligament ruptures, but the internal organs in this model were fused to form continuum bodied with homogeneous material properties, which means that the internal organs are not modeled individually. In order to extend the predictable range of the model, the research team refines the brain and internal organs structure for these issues [52]. The THUMS Ver.2.0 model had individual internal organs which include the bronchus, trachea, lung, heart, diaphragm, kidney, aorta, vena cava, spleen, esophagus, lung, stomach, pancreas, intestine, liver, and duodenum. These individual organs constituted the respiratory system, circulatory system, and digestive system. As for the brain model, a 2D head/brain model was developed, and they concluded that modeling of sulci of the cerebrum can affect the prediction of occurrence of brain injury. Then in 2007, THUMS Ver.3.0 model with a 3D brain consisting of the skull, brain, and skin was developed; the white matter, grey matter, cerebral spinal fluid (CSF), cerebellum, and cerebrum were included. The head/brain model was validated against three series of test data, in which translational and rotational accelerations were applied to the center of gravity (CG) of the head [53]. Then in 2012, the THUMS Ver.3.0 was mainly improved in the following aspects [54]: the model added some detailed parts, such as internal organs and the long bone in the lower extremities. In addition, the muscles had been added in the whole body, even in the sophisticated parts such as shoulder, chest, and lumbar spines. Moreover, the gap between the skull and the brain was eliminated at the base of the skull to more accurately represent the anatomy of the head and brain. These features had been verified by comparing the response with cadaveric and volunteer tests data from previous reference [55–57]. The updated THUMS with a vehicle sled model was used to investigate that the muscle activation levels and the activation timings had a nonnegligible effect on the driver's kinematics and injury outcomes. The updated THUMS is a promising tool to be used in accident injury reconstruction. In order to meet the need of real-world automotive accidents prediction, factors including body sizes, ages, and genders are considered by the research team. Therefore, a small 5th percentile female THUMS model [58] and a 6-year-old child THUMS [59] were developed successively as shown in Figure 2.

**Figure 3:**
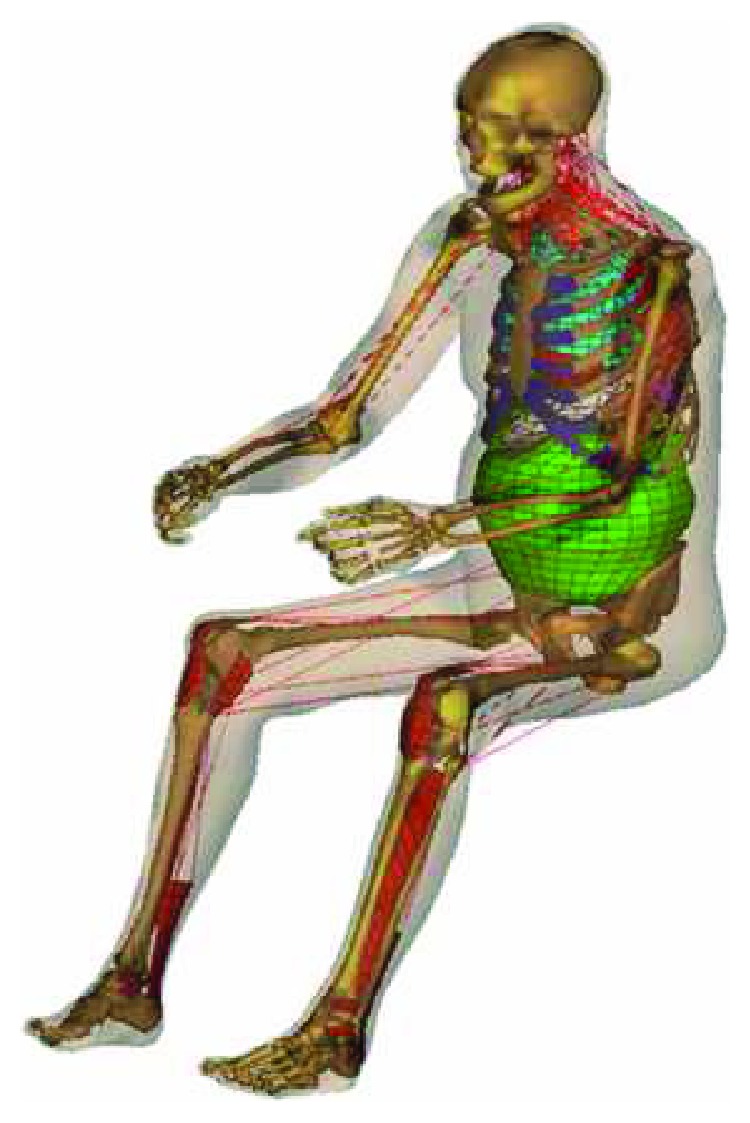
HUMOS model [66].

Since 1999, HUMOS (shown in Figure 3) was launched and funded by the European Commission in the Industrial and Materials Technologies (IMT) program (Brite–EuRam III), and the LAB (Laboratory of Accidentology and Biomechanics PSA Peugeot Citroën Renault) was involved in shoulder and the thorax meshing process [60]. Aiming at developing an exquisite human model that could be widely accepted by the crashworthiness community, the geometry acquisition is the basis of the task. By slicing a frozen cadaver, 491 images including detailed information of a 50th percentile European male were obtained. After the process of 3D geometrical reconstruction and meshing, the segment of the model had been validated by comparing the results to reference [13, 61–63]. Then further investigation on how muscular tensing influences the body response had been conducted by volunteer experiment [63]. HUMOS model had been validated having the ability to predict cervical trauma and other type trauma as well [64]. The human body was modified to study the relationship between chest deformation and the number of rib fractures. However, the results show that the maximum peak strain of the ribs does not correctly predict the number of rib fractures [65, 66].

**Figure 4:**
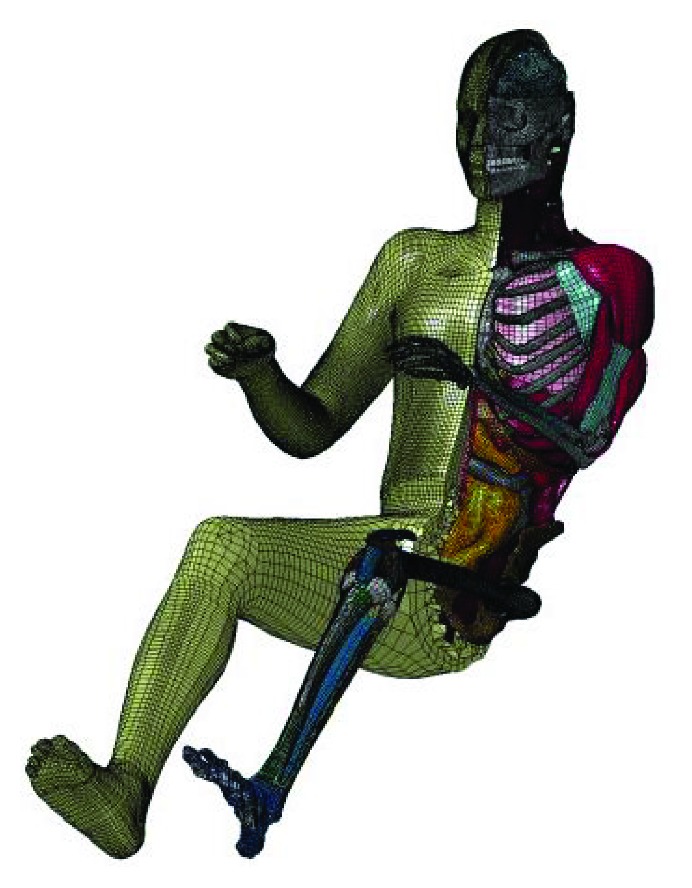
GHBMC model [68].

Committed to creating the world's most biofidelic computational human body model, the Global Human Body Models Consortium (GHBMC) developed a full-body CAD model of 50th percentile male model, which was called the GHBMC model (as shown in Figure 4). Gayzik et al. [67, 68] described the human data acquisition and model building process of a living 26-year-old male occupant (174.9 cm, 78.6 kg, BMI: 25.7) in detail. Seventy-two scans were performed using three medical imaging modalities (CT, MRI, and upright MRI); more than 300 individual components like bones (without thin cortical bone structures), organs (head, thorax, abdomen, etc.), vessels (without thin-wall vessels), muscles, cartilage, fibrocartilage, ligament, and tendon (without tissues) were generated through segmentation to represent the human anatomy. The model was validated from the component level, including the abdomen [69], cervical spine [70, 71], foot and ankle [72], and head [73]. And then whole-body validation had been conducted, under far-side conditions, Katagiri et al. [74] verified that the whole-body response of the GHBMC model had kinematic behavior sensitivity compared to six PMHS tests data [75], involving several parts such as the shoulder, head, pelvis, and abdomen. Under lateral sled and lateral drop conditions, Vavalle et al. [76] evaluated the whole-body response of the GHBMC model in thorax, abdomen, and pelvis regions and found that thorax and abdomen regions showed a good biofidelity. Park et al. [77] compared impact forces and kinematics data of GHBMC to that of PMHS obtained by Shaw et al. [78] at an impact velocity of 4.3 ± 0.1 m/s and assessed the biofidelity of GHBMC through correlation analysis. From the results, it can be concluded that the shoulder of the GHBMC model has a poor correlation with the PMHS, which means that the shoulder area needs to be improved. In order to improve the shoulder region, two modifications about material property of shoulder-related muscles and adipose tissue and three kinds of improvements on modeling technology were introduced into the repositioned model by Park et al. [79]; the sensitivity analysis showed that these modifications significantly influence the response and the shoulder region of modified model showed a better biofidelity. The research also indicated that the appropriate initial posture of the model contributes to fewer errors of peak shoulder deflection. Other researcher also realized the importance of initial posture on model biofidelity, and some research on repositioning were conducted. Marathe et al. [80] proposed a spline-based technique to locate the sagittal plane of human model; based on this research, different cubic splines are provided at the cervical, thoracic, and lumbar spine of GHBMC model by Chhabra [81], and the shape can be better controlled to predict the flexion, abduction, and twisting of human body by moving the control points. Chawla et al. [82] applied contour-based deformation technique to lower limbs (including ankle joint, knee joint, and hip joint) of the GHBMC model. Nonintersecting contours outline important skeleton; Delaunay triangulation method was then used to divide a three-dimensional space into small tetrahedrons, and the last step involves contour transformation based on the desired input, and it is expected that the key points can be transformed using the same parameters. This technology can greatly increase computational efficiency and ensure the calculation accuracy at the same time. The above studies are almost about the 50th percentile male model; in fact, establishing 5th percentile female GHBMC model was also listed as part of the project; the process of medical imaging dataset acquisition and the CAD model establishment was the same as that of the male model. The initial version of 5th percentile female had been established [83], but more validation work is needed in the future study.

It can be seen from the development of these human models that models are developing in the direction of gradual complication and anthropomorphization. However, with the refinement of the model mesh and the increase of the cells, the calculation time became longer and longer. Moreover, almost all of the existing human models are designed based on European and American men, which has limitation to predict the car crash injury for people of different genders, different countries, and different physical characteristics.

## 6. Conclusion

In this article, the development and verification process of mechanical dummies, related finite element models, and human models developed based on injury biomechanics are introduced in detail. From the description above, it can be seen that

1. the existing commercial crash test dummies are based on the human characteristics of Europe and the United States. From the perspective of injury mechanisms, they cannot represent the general human characteristics of other countries. In order to better protect the safety of occupants and improve the accuracy of injury prediction, each country should work hard to develop a crash test dummy that meets the human characteristics of its national conditions
2. most of the existing dummies and models are based on the male body. However, in the real collision, the elderly, obese, and dwarf women are more vulnerable to injury. In the subsequent model establishment, the diversity of people's type, size, and age can be taken into consideration

## Figures and Tables

**Table 1:** Abbreviated injury score.

AIS code	Injury severity	AIS% prob. of death
1	Minor	0
2	Moderate	1–2
3	Serious	8–10
4	Severe	5–50
5	Critical	5–50
≥6	Unsurvivable	100

**Table 2:** Common injury indicators for different parts of the human body.

Head	Neck	Chest	Abdomen	Pelvis and lower extremities
Acceleration (g) HIC value	Force (N) Force moment (N^∗^m) *N*_*ij*_ value	Deformation (mm) Acceleration (g) CTI value	Force (N)	Force (N)

**Table 3:** Dummies and their application areas.

Model name	Hybrid III	THOR-M	SID	SID-IIs
Figure	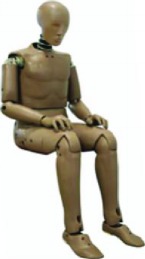	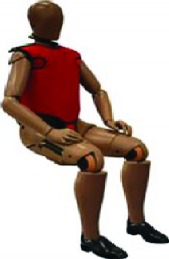	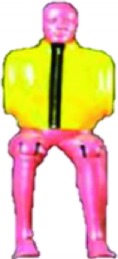	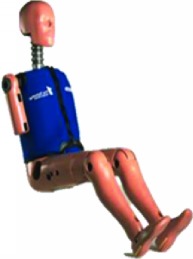
Application	Frontal impact	Frontal impact	Side impact	Side impact
Model name	BioSID	EuroSID-II	WorldSID	BioRID
Figure	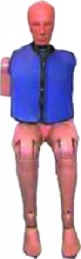	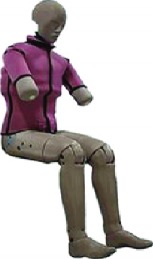	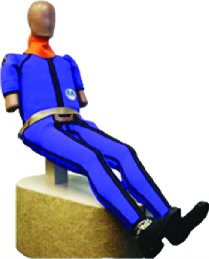	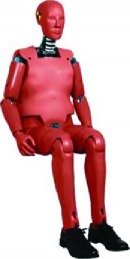
Application	Side impact	Side impact	Side impact	Rear impact

**Table 4:** ISO biofidelity classifications.

Level	Excellent	Good	Fair	Marginal	Unacceptable
Score range	>8.6 to 10.0	>6.5 to 8.6	>4.4 to 6.5	>2.6 to 4.4	0 to 2.6

**Table 5:** Side impact dummy biofidelity comparison.

	Biofidelity rating						
	Head	Neck	Shoulder	Chest	Abdomen	Pelvis	Overall
WorldSID	10	5.3	10	8.2	9.3	5.1	8
BioSID	6.7	6.7	7.3	6.3	3.8	4	5.7
EuroSID-I	5	7.8	7.3	5.4	0.9	1.5	4.4
ES-II	5	4.4	5.3	5.2	2.6	5.3	4.6
